# Tumeur de Frantz: deux nouveaux cas

**DOI:** 10.11604/pamj.2013.14.7.1412

**Published:** 2013-01-04

**Authors:** Salma Bellarbi, Mohamed Sina, Ahmed Jahid, Fouad Zouaidia, Zakia Bernoussi, Najat Mahassini

**Affiliations:** 1 Service Central D'anatomie Pathologique et Cytologie, CHU Ibn Sina, Rabat, Maroc

**Keywords:** Tumeur de Frantz, pancréas, tumeur pseudopapillaire, tumeur solide, kyste, Frantz's tumor, pancreas, pseudopapillary tumor, solid tumor, cyst

## Abstract

A travers cet article, nous détaillons les caractéristiques clinico-pathologiques et discutons l'histogenèse de la tumeur de Frantz. Deux patients opérés pour tumeur de Frantz. Ils ont eu un traitement chirurgical seul. L'étude morphologique était couplée à un examen immuno-histochimique (IHC) utilisant les anticorps anti CD10, anti- vimentine, anti-énolase neuronale spécifique (NSE), anti-synaptophysine, anti-chromogranine A et anti-cytokératine. Un immuno-marquage à l'anti-oestrogène et l'anti-progestérone a été réalisé dans un cas. Il s'agissait d'une femme âgée de 45ans et d'un garçon de 12 ans. Les aspects échographiques et scannographiques étaient non spécifiques. Une exérèse chirurgicale complète a été réalisée dans les deux cas. L'analyse histologique évoquait une tumeur de Frantz. Le diagnostic a été retenu après étude immuno-histohimique. L'évolution était favorable sans récidive avec respectivement un recul de 18 et 16 mois. La tumeur de Frantz est une entité rare. Son diagnostic repose sur l'examen anatomopathologique complété par l'étude immuno-histochimique. Son pronostic est excellent après résection chirurgicale.

## Introduction

Décrite pour la première fois en 1959, la tumeur de Frantz ou tumeur pseudopapillaire solide et kystique du pancréas (TPPSP) est classée par l'Organisation Mondiale de la Santé comme une tumeur à potentiel malin incertain [1] et représente 1 à 2% de toutes les tumeurs exocrines du pancréas [2]. Elle touche avec prédilection la femme jeune, rarement l'homme et l'enfant. La symptomatologie clinique est non spécifique et aucune anomalie biologique n'est retrouvée. Les explorations radiologiques montrent habituellement une masse bien limitée, peu vascularisée [3]. Son diagnostic positif est anatomopathologique et son traitement est chirurgical. Son pronostic est excellent après résection complète. Nous rapportons deux nouveaux cas de tumeur de Frantz, en détaillant ses caractéristiques clinico-pathologiques et discutant son histogenèse.

## Patient et observation

Deux patients, opérés en 2010 pour tumeur de Frantz. Les données cliniques, paracliniques et évolutives étaient recueillies à partir des dossiers médicaux. L'étude morphologique était couplée à un examen immuno-histochimique (IHC) utilisant les anticorps anti CD10, anti-vimentine, anti-énolase neuronale spécifique (NSE), anti-synaptophysine, anti-chromogranine A et anti- cytokératine. Un immuno-marquage à l'anti-oestrogène et l'anti-progestérone a été réalisé dans un cas.

Il s'agissait d'une femme âgée de 45 ans et un garçon de 12 ans. Les caractéristiques cliniques, paracliniques et chirurgicales sont détaillées dans le Tableau 1. Les deux cas ont eu un traitement chirurgical seul.

**Tableau 1:** Caractéristiques cliniques, paracliniques et chirurgicales de nos patients

Sexe	Age (ans)	Circonstances de découverte	Imagerie	Diagnostic évoqué	Localisation	Diametre (cm)	Intervention chirurgicale	Recul (mois)
Féminin	45	Fortuite (échographie pour aménorrhée)	Echographie TDM	-	Céphalique	8	DPC (duodénopancréatectomie céphalique)	18
Masculin	12	Douleurs abdominales	Echographie TDM	-	céphalique	5	DPC	16

**Figure 1:**
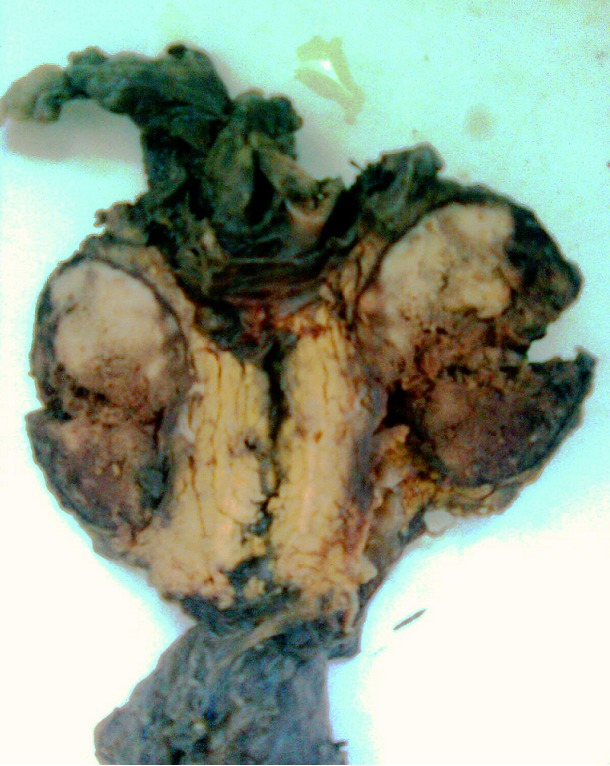
Aspect macroscopique de la tumeur de Frantz de localisation céphalique. Tumeur bien limitée avec remaniements nécrotico-hémorragiques

**Figure 2:**
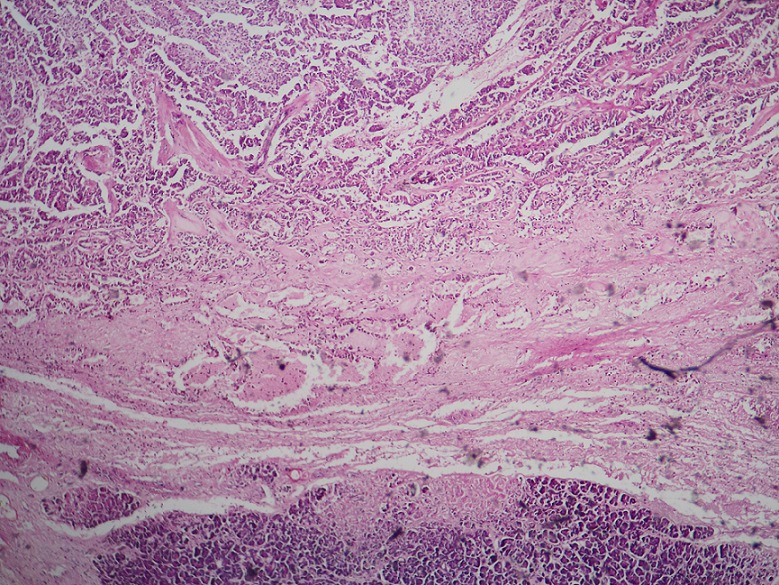
Capsule fibreuse séparant la tumeur du pancréas normale en bas (HEx4)

**Figure 3:**
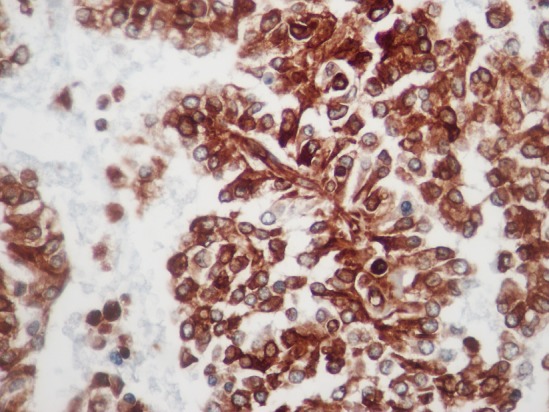
Immuno-marquage cytoplasmique des cellules tumorales à l'anti-CD10 (HEx40)

**Figure 4:**
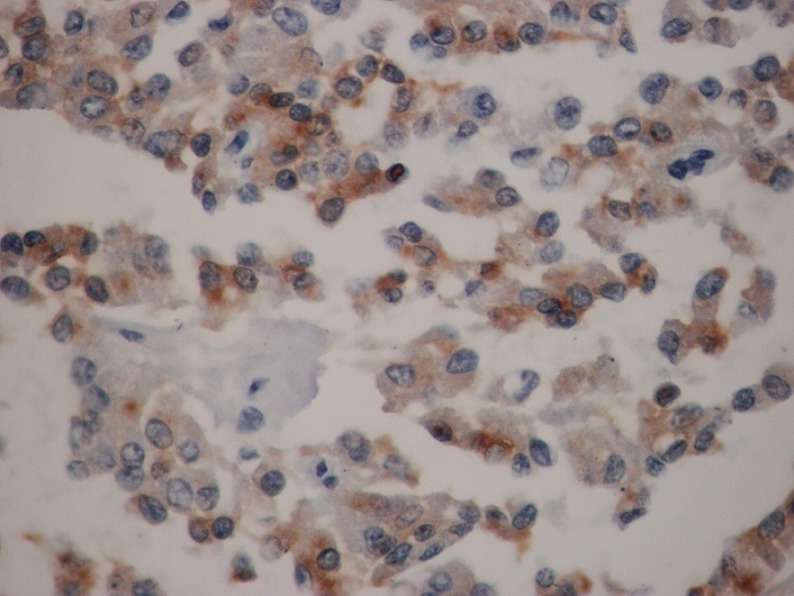
Immuno-marquage cytoplasmique des cellules tumorales à l'anti-vimentine (HEx40)

L'examen macroscopique des pièces opératoires retrouvait des masses tumorales bien limitées, encapsulées, renfermant souvent des remaniements kystiques et hémorragiques (Figure 1). Histologiquement, il s'agissait d'une prolifération faite de nappes diffuses et de structures pseudopapillaires; les cellules tumorales étaient assez monomorphes, cuboïdes ou polygonales de petite taille, au cytoplasme clair parfois vacuolisé. Le noyau cellulaire était peu atypique sans figures de mitoses (Figure 2). Les marges d'exérèse chirurgicale étaient saines. Les résultats de l'examen immuno-histochimique sont détaillés dans le Tableau 2, Figure 3, Figure 4. Le diagnostic retenu était celui de tumeur de Frantz. L'évolution était favorable sans récidive avec respectivement un recul de 18 et 16 mois.

**Tableau 2:** Résultats de l'examen immuno-histochimique de nos patients

	Cas	
Anticorps	femme	garçon
CD10	+ (Figure 4)	+
vimentine	+ (Figure 5)	+
NSE	+	+
synaptophysine	Faible, focale	-
chromogranine	Faible, focale	-
cytokératine	-	-
œstrogène	-	Non fait
progestérone	+	Non fait

## Discussion

La tumeur de Frantz ou TPPSP est une affection rare (1 à 2% des tumeurs exocrines du pancréas), de malignité atténuée qui s'observe dans plus de 95% des cas chez la femme jeune [4]. La tumeur se situe le plus souvent au niveau de la queue du pancréas (40% des cas). Sa taille varie de 2,5 à 25 cm de grand axe. La tumeur est arrondie ou ovalaire, limitée par une capsule fibreuse épaisse. Elle est le siège de remaniements nécrotiques et hémorragiques, réalisant un aspect pseudokystique assez caractéristique [5], comme dans nos cas. Le diagnostic positif est histologique. La tumeur est constituée de plages solides périphériques et de structures papillaires centrales. Les cellules tumorales sont monomorphes, de petite taille, cuboïdes ou polygonales et souvent agencées autour de septa fibrovasculaires. Les mitoses et les atypies cytonucléaires sont exceptionnelles. On peut trouver des amas d'histiocytes spumeux et des cellules géantes autour de cristaux de cholestérol. Le stroma est habituellement de type endocrine, riche en capillaires sanguins. Les critères anatomo-pathologiques de malignité sont retrouvés dans seulement 10 à 15% des cas (envahissement des structures adjacentes, emboles vasculaires, invasion périnerveuse et métastases ganglionnaires ou à distance); dans ces cas, la TPPSP est classée carcinome pseudopapillaire et solide [1]. Le profil immuno-histochimique de la TPPSP est variable. Habituellement, les cellules tumorales sont marquées par les anticorps anti-CD 10, alpha-1- antitrypsine, vimentine, NSE, E-cadérine et bêtacaténine. On note aussi un marquage à l'anticorps anti-progestérone [6]. L'immunomarquage positif des cellules tumorales pour certains marqueurs endocrines peut attester d'une certaine différenciation endocrine [3].

Le diagnostic différentiel se pose essentiellement chez l'adulte avec l'adénocarcinome canalaire du pancéars, les tumeurs neuroendocrines et les pseudokystes du pancréas; cependant les caractéristiques macroscopiques et l'aspect histologique de la TPPSP rendent le diagnostic différentiel facile. L'adénocarcinome canalaire se rencontre volontiers chez les hommes âgés et la tumeur est plus petite. Histologiquement, elle est composée de structures tubulaires et glandulaires évoluant de façon infiltrante.

Les tumeurs endocrines montrent usuellement des aspects solides et microacinaires; des zones hémorragiques et pseudokystiques sont rarement observées. Sur le plan cytologique, les noyaux sont petits, ronds avec une chromatine fine. L'étude IHC montre un marquage diffus et intense par les anticorps anti-NSE, anti-chromogranine et anti-synaptophysine.

La TPPSP peut être confondue macroscopiquement avec un pseudokyste du pancréas mais sur le plan histologique le diagnostic est facilement établi [7]. Chez l'enfant, le diagnostic différentiel se pose avec le pancréatoblastome. Ce dernier se voit habituellement avant l'âge de 8 ans. Sa structure est mixte, solide, trabéculaire et kystique. Il existe des corpuscules de cellules squameuses et du tissu mésenchymateux caractéristique [8].

L'étiopathogénie de la tumeur de Frantz reste controversée. L'expression des récepteurs à la progestérone et la prédilection pour le sexe féminin suggèrent l'hormono-dépendance de cette tumeur. Une origine centro-acineuse a été également avancée, en se basant sur des constatations immuno-histochimiques et ultrastructurales [9]. Cependant, ce domaine requiert de nouvelles investigations afin de valider ces théories.

Sur le plan thérapeutique, seule l'exérèse chirurgicale est associée à un pronostic favorable avec une survie post-opératoire de 80 à 90%; le taux de récidive est de 10 à 15% [10].

## Conclusion

La tumeur de Frantz représente moins de 2% des tumeurs exocrines du pancréas. Elle touche préférentiellement la femme jeune. La symptomatologie clinique est non spécifique. Les explorations radiologiques orientent le diagnostic et l'étude anatomopathologique permet de le confirmer. L'étude immuno-histochimique aide à éliminer les tumeurs d'autre nature. L'étiopathogénie de ces tumeurs reste controversée.
